# Judicial assessment of testimonial reliability after EMDR therapy: a case-series analysis

**DOI:** 10.3389/fpsyg.2026.1809865

**Published:** 2026-06-02

**Authors:** Federico Pacchioni, Emma Flutti, Marta Ballabio, Marie Emilie Giovannelli, Caterina Magatti, Guido Travaini

**Affiliations:** 1 Faculty of Philosophy, Vita-Salute San Raffaele University, Milan, Italy; 2 Mood Disorder Unit, IRCCS San Raffaele Hospital, Milan, Italy; 3 Faculty of Medicine, Vita-Salute San Raffaele University, Milan, Italy

**Keywords:** criminal court, EMDR, false memory, minors, sexual abuse, testimony, trauma therapy

## Abstract

**Introduction:**

Eye Movement Desensitization and Reprocessing (EMDR) is a first-line psychotherapy for post-traumatic stress disorder. Despite its established clinical efficacy, concerns have been raised regarding its potential effects on autobiographical memory and testimonial reliability in judicial contexts. Given the reconstructive nature of memory, this study aimed to examine how Italian courts address EMDR when evaluating witness credibility.

**Methods:**

A targeted jurisprudential analysis was conducted using the DeJure legal database. Final decisions of the Italian Supreme Court (Corte di Cassazione) issued up to January 2026 were retrieved and screened to identify rulings in which EMDR therapy was explicitly mentioned and played a forensically relevant role in the assessment of testimonial credibility, memory reliability, or evidentiary reasoning. Eligible cases were required to constitute final, non-appealable rulings and to include substantive judicial or expert discussion of EMDR. Two researchers independently screened, selected, and analyzed the decisions according to predefined inclusion criteria, applying a qualitative case-series approach.

**Results:**

Five Supreme Court decisions met the inclusion criteria, all involving criminal proceedings with minor victims and credibility assessment. EMDR was primarily raised by the defense as a potential source of memory alteration. The Court consistently rejected categorical assumptions of reduced testimonial reliability. Instead, it required concrete, case-specific evidence of memory distortion. Where credibility was questioned, judicial criticism focused on procedural and methodological deficiencies, particularly inadequate documentation, and insufficient separation between therapeutic and investigative roles.

**Discussion:**

Italian Supreme Court jurisprudence reflects a cautious and context-sensitive approach to testimony following EMDR. The therapy is recognized as clinically legitimate and is not considered intrinsically suggestive. Judicial concerns are directed toward procedural safeguards and transparency rather than the therapeutic technique itself. These findings support a case-by-case evaluation model and highlight the importance of rigorous clinical documentation and role differentiation in forensic settings.

## Introduction

Human memory is not a static repository of past events, but a dynamic and reconstructive process shaped by cognitive, emotional, and contextual factors (Craik, 2020; Lacy and Stark, 2013).

At the same time, traumatic memory cannot be reduced exclusively to ordinary autobiographical reconstruction. Trauma-related memories may also emerge as intrusive, sensory, and affectively charged experiences, including flashbacks and involuntary recollections. These phenomena may differ from structured autobiographical recall, as they can be experienced as vivid, present-oriented re-experiencing rather than as narratives of past events (Brewin, 2007; Van der Kolk and Fisler, 1995).

Contemporary cognitive science demonstrates that recollection involves active reconstruction rather than passive replay, and that accuracy, vividness, and subjective confidence may dissociate, particularly following repeated retrieval or exposure to external information (Loftus, 2003; Otgaar et al., 2022a).

Memory reconsolidation, whereby reactivated memories become temporarily labile and susceptible to modification before restabilization, represents a central mechanism through which memories may be updated, strengthened, or distorted over time (Bellfy and Kwapis, 2020; Lee et al., 2017; Schwabe et al., 2014). These characteristics are especially consequential in legal contexts, where fact-finding frequently relies on narrative recall under conditions that facilitate post-event updating and source confusion (Greene et al., 2025; Lacy and Stark, 2013; Loftus, 2003; Otgaar et al., 2023). The misinformation effect has further challenged the reliability of eyewitness testimony, although its underlying mechanisms remain debated (Brassil et al., 2024; Wiechert et al., 2025).

The fragility of autobiographical memory in forensic psychology, particularly in eyewitness testimony, is well documented. Recent research indicates that both children and adults are vulnerable to false memory formation through post-event information, suggestive questioning, emotional arousal, and source-monitoring errors (Otgaar et al., 2021; Pérez-Mata and Diges, 2024). Experimental paradigms show that it is often difficult to distinguish reliably between true and false memories when testimony is the sole evidence, and that suggestive practices may influence both minors and adults (Pérez-Mata and Diges, 2024). Empirical reviews further confirm the role of memory distortion in wrongful convictions, especially in cases involving historic abuse and eyewitness misidentification (Howe and Knott, 2015).

In vulnerable populations, including children and individuals with psychological distress, suggestibility is heightened, with complex developmental patterns indicating that, under certain conditions, adults may also be highly susceptible to false memories (Otgaar et al., 2018; Rosendaul et al., 2023). In Italy, the Carta di Noto IV (2017) guidelines emphasize memory malleability and the need to prevent contamination through suggestive or undocumented interviews, in line with international best practices (Montanari Vergallo et al., 2018).

Dissociative processes may further complicate the relationship between trauma and memory. In traumatic contexts, dissociation may affect encoding, consolidation, and retrieval, potentially resulting in fragmented, poorly integrated, or temporarily inaccessible autobiographical material. Importantly, dissociation does not imply either accuracy or inaccuracy per se; rather, it highlights the need to evaluate how, when, and under what conditions traumatic memories are accessed, narrated, and documented (Van der Kolk and Fisler, 1995; Brewin, 2007).

The “memory wars” of the 1990s initially framed around two contrasting positions on recovered memories of childhood abuse. One perspective supported the existence of repression or dissociative amnesia, allowing for accurate recovery in therapy (Battista et al., 2023; Otgaar et al., 2019; Patihis et al., 2014). The opposing view, grounded in experimental research, highlighted the risks associated with suggestive practices, which may foster the formation of false autobiographical memories (Geraerts et al., 2009; Otgaar et al., 2022a).

Legal and scientific analyses have underscored the risk of unfounded accusations and miscarriages of justice in the absence of independent corroboration (Otgaar et al., 2019; Otgaar et al., 2022b). Current consensus recognizes that recovered memories may be genuine, false, or mixed, and that attempts to exhume memories represent a significant methodological risk (Battista et al., 2023; Brewin, 2021; Dodier et al., 2024; Otgaar et al., 2019; Otgaar et al., 2022b). This perspective reflects a shift away from strictly polarized interpretations toward a more integrative understanding of memory processes.

This evolution is reflected in more recent theoretical models of traumatic memory, which move beyond strictly polarized interpretations (Otgaar et al., 2022b). Earlier debates on traumatic memory, often referred to as the “memory wars,” were historically framed around opposing views on repression, dissociative amnesia, recovered memories, and false memories. However, contemporary literature has moved beyond a strictly dichotomous account (Brewin, 2007; Brewin, 2021). Current research recognizes that traumatic memories may be retained, avoided, fragmented, forgotten, later recalled, or reconstructed, depending on the interaction between individual vulnerability, developmental factors, emotional arousal, dissociation, retrieval conditions, and social or therapeutic context (Brewin, 2007; Van der Kolk and Fisler, 1995).

Accordingly, memories that emerge or are elaborated in therapy should not be treated as either inherently reliable or inherently unreliable. Rather, their forensic significance depends on the conditions under which they were retrieved, narrated, reinforced, and documented (Selaya et al., 2023).

Clinical and neurobiological models of trauma further emphasize that traumatic experiences may involve altered integration between emotional, sensory, bodily, and narrative components of memory. Neuroimaging studies have implicated cortical, limbic, and thalamic networks in trauma-related autobiographical memory and PTSD, suggesting that traumatic recall may be shaped by altered arousal, emotional processing, and memory integration (Hughes and Shin, 2011; Kearney and Lanius, 2022; Thome et al., 2020).

Among evidence-based psychotherapies for trauma, Eye Movement Desensitization and Reprocessing (EMDR) has attracted particular attention for both its clinical efficacy and its implications for memory processing. Developed by Francine Shapiro in 1987 for the treatment of post-traumatic stress disorder (PTSD), EMDR is grounded in the Adaptive Information Processing (AIP) model, which posits that psychopathology arises from inadequately processed traumatic memories stored in a dysfunctional manner. The treatment aims to promote adaptive reprocessing and integration into broader, non-distressing cognitive networks (Hase et al., 2025; Hase and Brisch, 2022; Shapiro and Maxfield, 2002).

A central component of EMDR is bilateral stimulation, most commonly through guided eye movements, but also via alternating auditory or tactile stimuli (Calancie et al., 2018; Ross et al., 2017). During sessions, patients recall distressing memories while simultaneously engaging in bilateral stimulation, following a dual-attention procedure that taxes working memory, reduces vividness and emotional intensity, and facilitates memory integration (Jeffries and Davis, 2013; Landin-Romero et al., 2018). Meta-analytic evidence supports the specific contribution of eye movements compared with protocols lacking this component (American Psychological Association, 2017).

More than thirty randomized controlled trials have demonstrated the effectiveness of EMDR in adults and children, with significant reductions in PTSD symptoms, depression, and anxiety (Chen et al., 2014; de Jongh et al., 2024; Rasines-Laudes and Serrano-Pintado, 2023; Wilson et al., 2018). Meta-analyses indicate outcomes comparable to trauma-focused cognitive behavioral therapy, with shorter treatment duration and lower patient burden (Hoppen et al., 2023; Hudays et al., 2022; Simpson et al., 2025; Wright et al., 2024). Accordingly, clinical guidelines from NICE and WHO recommend EMDR as a first-line intervention for PTSD (de Jongh et al., 2024; Schnurr et al., 2024).

The working memory model suggests that dual-attention procedures may reduce the vividness and emotional intensity of traumatic memories, although findings concerning their effects on memory accuracy and false memory formation remain mixed and methodologically debated (Landin-Romero et al., 2018; Mertens et al., 2021; Wadji et al., 2022).

Experimental studies have reported higher rates of such phenomena, particularly after delayed retrieval, without consistent increases in susceptibility to external suggestion (Houben et al., 2020; Otgaar et al., 2021; Otgaar and Houben, 2026). However, subsequent research has yielded null or inconsistent effects on recognition tasks, limiting generalizability (Aleksic et al., 2025). These findings suggest the need for cautious and context-sensitive forensic evaluations of post-treatment testimony, particularly when memory retrieval procedures and clinical documentation are unclear.

Recent literature indicates that, although EMDR may affect memory quality, it does not systematically increase suggestibility (Otgaar and Houben, 2026). From a forensic perspective, potential risks appear to stem primarily from procedural and contextual factors, including role confusion, inadequate documentation, and insufficient separation between therapeutic and investigative aims (Otgaar et al., 2021; Otgaar et al., 2022a). Consequently, concerns about testimonial reliability call for case-by-case assessments grounded in methodological transparency and reconstruction of narrative formation, rather than categorical assumptions.

Despite increasing scientific debate, empirical analyses of judicial approaches to EMDR remain scarce, as the literature is predominantly experimental or clinical (Otgaar et al., 2022a; Otgaar et al., 2021; Otgaar and Houben, 2026). Only one case-based contribution has examined Italian jurisprudence, limited to a single decision (Otgaar et al., 2022b), a gap that is particularly relevant given the specific features of the Italian legal system and the Carta di Noto IV guidelines (Montanari Vergallo et al., 2018).

To address this gap, the present study provides a qualitative jurisprudential case-series analysis of final decisions issued by the Italian Supreme Court (Corte di Cassazione) in which EMDR is explicitly mentioned, examining how testimonial credibility is assessed. By integrating scientific evidence with judicial practice, this article aims to offer an original contribution to forensic psychology and legal scholarship, with practical implications for clinicians and expert witnesses.

## Materials and methods

A targeted jurisprudential analysis was conducted to identify the Supreme Court decisions in which EMDR therapy is explicitly mentioned. Retrieval was performed through DeJure, a comprehensive Italian legal database providing access to published case law.

The search was conducted in Italian using the DeJure legal database and covered all available years up to January 2026. The following search string was used: (“EMDR” OR “Eye Movement Desensitization and Reprocessing”) AND (“testimony” OR “witness” OR “credibility”). Additional manual screening was performed to identify decisions in which EMDR was discussed in relation to testimonial reliability or evidentiary reasoning.

### Eligibility criteria

Decisions were deemed eligible if they met all the following criteria:

- The decision was issued by the Supreme Court, thus constituting a final ruling (i.e., no longer subject to appeal);^1^
- EMDR was explicitly mentioned in the reasoning of the decision or in cited expert opinions (e.g., court-appointed or party-appointed expert reports);
- The reference to EMDR was forensically relevant, insofar as it had evaluative, probative, or argumentative significance for the assessment of testimony, memory reliability, mental capacity, or related evidentiary issues;
- The full text of the decision was accessible through DeJure or other public, official, and legal sources.

Decisions were excluded when EMDR was mentioned only descriptively (e.g., as part of clinical background), without any evaluative relevance for testimonial credibility, memory reliability, or evidentiary reasoning.

### Screening and data extraction

All retrieved records were independently reviewed by two researchers with expertise in forensic psychopathology and legal analysis. Full texts were examined for eligibility, and interpretative divergences concerning inclusion or case interpretation were resolved through discussion and consensus.

Included decisions were analyzed using a qualitative jurisprudential case-series approach focused on the interpretative examination of judicial reasoning concerning testimonial credibility and the role attributed to EMDR within evidentiary assessment.

The analysis focused on recurrent elements within judicial reasoning, including the procedural context of the case, the role attributed to EMDR within the proceedings, arguments concerning memory reliability, and the criteria used by the courts to assess testimonial credibility.

Although the study does not follow a systematic review design, the search and selection process was structured to enhance transparency and replicability, by explicitly defining search criteria, eligibility requirements, and screening procedures.

The aim of this approach was not merely descriptive, but to provide an interpretative analysis of judicial reasoning by identifying common themes and divergences across cases.

## Results

The targeted jurisprudential search yielded eight judicial decisions. Following full-text examination, three decisions were excluded because EMDR was referenced only incidentally and without any substantive relevance to evidentiary reasoning or testimonial assessment.

**Figure 1:**
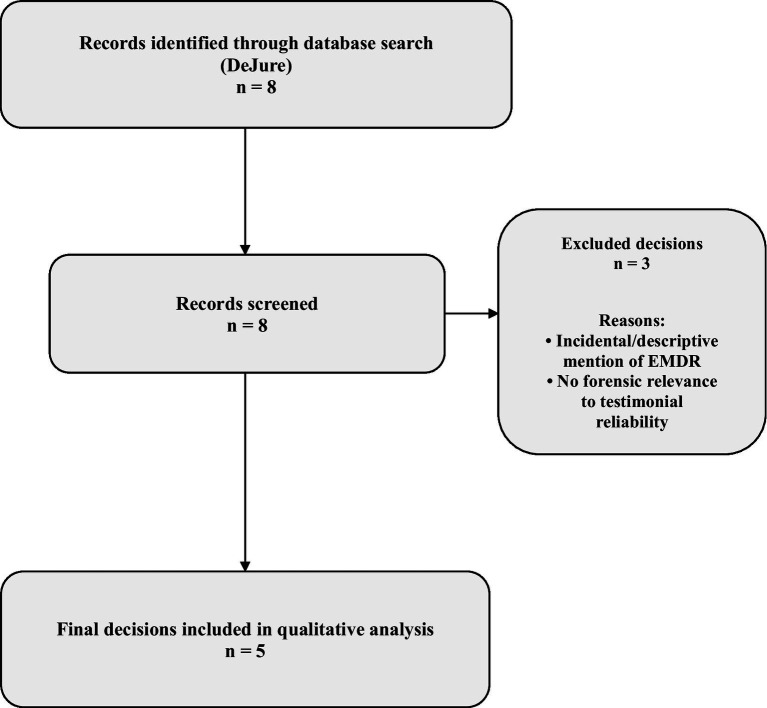
Flow diagram of the selection process for judicial decisions included in the qualitative jurisprudential case-series analysis.

A flow diagram summarizing the search and selection process is presented in Figure 1.

The final analytical corpus consisted of five eligible decisions issued by the Italian Supreme Court, all concerning criminal proceedings involving minor victims and centering on the assessment of testimonial credibility for the establishment of criminal responsibility.

### Characteristics of the included decisions

The main characteristics of the included decisions are summarized in Table 1. Extracted information comprised:

- The decision reference the procedural context of the case;
- The procedural role attributed to EMDR, whether as part of the therapeutic background, as an element contested by the parties, or as the object of forensic or expert evaluation;
- The judicial assessment of EMDR in relation to testimonial credibility and the evidentiary arguments advanced by defense counsel and expert witnesses;
- The outcome of the judgment.

**Table 1:** Characteristics of the included decisions.

Decision (Supreme Court)	Procedural context	Role of EMDR	Judicial reasoning theme	Outcome
Italian Supreme Court, Criminal Section III, No. 5234/2022	Criminal, Ordinary Court	Defense argued that pre-hearing EMDR reduced emotional intensity and impaired reliability	Rejection of abstract EMDR-related unreliability. Requirement of case-specific evidence	Conviction (partially reformed on appeal); appeal declared inadmissible
Italian Supreme Court, Criminal Section III, No. 1193/2022	Criminal, Juvenile Court	EMDR shortly before testimony alleged to affect credibility	Confirmation of testimonial credibility in the absence of concrete evidence of EMDR-related influence.	Conviction confirmed; appeal dismissed
Italian Supreme Court, Criminal Section III, No. 10068/2020	Criminal, Ordinary Court	Therapist first recipient of disclosure; EMDR prior to testimony	Focus on procedural flaws (documentation, role confusion); no intrinsic prejudice attributed to EMDR	Annulment with remand for renewed credibility assessment
Italian Supreme Court, Criminal Section III, No. 23933/2021	Criminal, Ordinary Court	Defense requested expert assessment due to prior EMDR	Rejection of expert assessment request. No concrete grounds linking EMDR to impaired testimony	Conviction confirmed; appeal dismissed
Italian Supreme Court, Criminal Section V, No. 28087/2024	Criminal, Ordinary Court	Alleged misuse of EMDR to elicit previously unremembered events	Strict standard for causal inference; absence of scientific proof evidence linking EMDR to psychological harm.	Acquittal confirmed

### Judicial assessment of EMDR

The analysis focused on how EMDR was addressed within judicial reasoning and on whether it was considered capable of influencing testimonial reliability in the specific circumstances of each case. This qualitative, context-sensitive approach allowed for an examination of judicial decision-making that avoided rigid classifications and remained consistent with the exploratory nature of the study.

Across the examined decisions, EMDR emerged primarily as a contested element raised by the defense, reflecting the central evidentiary role of the minor’s testimony in the proceedings. Defense arguments typically framed EMDR as a potential source of memory alteration or suggestibility, while expert opinions and judicial reasoning differed in the weight attributed to such claims.

Accordingly, the results presented below focus not merely on the presence of EMDR within the procedural background, but on its actual or alleged interaction with testimonial evidence, as interpreted and evaluated by the Supreme Court. The following section provides a concise case-by-case account, outlining the procedural trajectory of each matter from the first-instance judgment to the final ruling.

### Cases analysis

#### Case I

*(Italian Supreme Court, Criminal Division III, Judgment no. 5234/2022)*

The proceedings concerned the offences of sexual acts with a minor, aggravated group sexual assault, and possession of child pornography. The defense raised a specific objection to the testimonial reliability of the complainant, emphasizing that the minor had undergone, prior to being heard, a therapeutic course that included the use of EMDR. According to the defense, this treatment had negatively affected the genuine processing of the events, compromising the reliability of the testimony. The trial court rejected this argument, holding that the abstract capacity of EMDR therapy to affect memories could not justify the exclusion or radical unreliability of testimonial evidence in the absence of concrete and specific elements demonstrating an actual alteration of memory.

On appeal, the defense reiterated the same arguments. The Court of Appeal upheld the assessment of credibility, highlighting the internal consistency, richness of detail, and narrative stability of the complainant’s account, as well as the absence of evidence capable of demonstrating, in the specific case, an alteration of memory processes attributable to the therapeutic treatment.

Before the Supreme Court, the defense renewed its position, but The Supreme Court dismissed this argument, stating that *“a perspective that assumes an automatically invalidating effect of EMDR therapy on the testimonial capacity of the complainant cannot be endorsed.”*

The Court clarified that *“the methodological error committed by the appellant lies in reasoning by abstractions: the issue is not whether, in the abstract, EMDR may alter a victim’s memories, but whether, in concrete terms, it actually did so.”*

The Court further emphasized that there is *“no certainty (nor scientific convergence) that EMDR techniques invariably and necessarily produce false memories.”*

In its reasoning, the Court reaffirmed that undergoing psychological therapy prior to giving testimony does not, per se, constitute a factor of automatic unreliability.

#### Case II

*(Italian Supreme Court, Criminal Division III, Judgment no. 1193/2022)*

The case concerned group sexual assault against a minor, committed by a juvenile offender acting in concert with an adult.

During appellate proceedings, defense counsel challenged the credibility of the complainant’s testimony, arguing that the minor’s prior engagement in a therapeutic pathway including EMDR, before being examined as a witness, had compromised the genuineness of the narrative.

The Court of Appeal rejected this objection, confirming the reliability of the complainant’s testimony. In particular, the judges noted the absence of concrete elements demonstrating an actual conditioning of mnemonic processes attributable to EMDR, as well as the coherence and overall consistency of the account throughout the proceedings.

The Supreme Court upheld the lower court’s reasoning, emphasizing that the assessment of testimonial reliability had been conducted in a detailed and rigorous manner.

#### Case III

*(Italian Supreme Court, Criminal Division III, Judgment no. 10068/2020)*

The proceedings concerned aggravated sexual assault against the defendant’s minor daughter. In the merit’s judgments, criminal liability was established primarily on the basis of the complainant’s statements, deemed credible. The minor had undertaken a therapeutic course involving EMDR, and the therapist was the first recipient of the disclosure of abuse. During the trial, the psychologist treating the minor testified as a witness.

On appeal, the defense challenged the credibility of the complainant, alleging potential contamination of memory and the formation of false memories, with specific reference to the use of EMDR in the therapeutic context. According to the defense, this circumstance should have prompted a more cautious and rigorous assessment of the victim-witness’s credibility.

The Court of Appeal rejected these arguments, finding the complainant’s statements credible and identifying no elements capable of demonstrating interference between the therapeutic pathway and the subsequent judicial testimony.

In the present case, the Court undertook an extensive examination of the proper modalities for the taking of witness testimony and of the need to clearly delineate the delicate role of the psychologist in the specific circumstances of the case. Such deficiencies led to the quashing of the appellate judgment. Notwithstanding the foregoing, in its reasoning the Supreme Court did not attribute to EMDR any automatically prejudicial effect on the credibility of the witness’s testimony.

#### Case IV

*(Italian Supreme Court, Criminal Division III, Judgment no. 23933/2021)*

The proceedings concerned acts of a sexual nature involving a minor. The court of first instance found the defendant guilty based on the complainant’s statements, which were deemed credible, together with corroborating factual elements.

On appeal, the defense challenged the credibility of the victim’s statements, arguing in particular for the necessity of an expert assessment of testimonial reliability, in light of the fact that the complainant had undergone EMDR therapy prior to testifying.

The Court of Appeal excluded the need for further evidentiary proceedings by way of an expert report, finding no concrete elements capable of justifying a technical evaluation of the witness’s testimonial capacity because of the EMDR therapy.

The Supreme Court upheld this approach. Although it did not directly address the impact of EMDR on memory, the Court implicitly ruled out the existence of any legally relevant correlation between the administration of such therapy and an impairment of testimonial capacity.

#### Case V

*(Italian Supreme Court, Criminal Division V, Judgment no. 28087/2024)*

The proceedings concerned a psychotherapist charged with aggravated bodily harm against a minor, allegedly committed in the exercise of professional activity. The defendant was accused of having caused the onset of borderline personality disorder and persistent depressive disorder with anxiety as a consequence of allegedly inappropriate therapeutic treatment.

At first instance, the court affirmed criminal liability, attributing relevance to the use of EMDR in the minor’s therapeutic pathway. According to the judgment, the technique had been applied in a manner inconsistent with established protocols, establishing a causal link between therapeutic error and the alleged harm, identified in the emergence of false memories of sexual abuse and the consequent psychological destabilisation of the complainant.

On appeal, the conviction was entirely overturned, and the psychotherapist was acquitted. The appellate court held that the prosecution’s expert opinion failed to adequately identify the scientific laws of coverage necessary to establish, under criminal causation standards, that the therapeutic conduct had caused the diagnosed psychological disorders.

The Supreme Court of Cassation confirmed the acquittal, finding the appellate reasoning coherent and consistent with principles governing criminal causation. The Court emphasised the absence of scientifically grounded proof of an etiological link between the contested therapeutic conduct and the alleged psychopathological outcomes. It reiterated that criminal liability for psychological injury cannot be based solely on temporal sequence but requires demonstration, through scientifically validated laws of causation, of the etiological nexus beyond reasonable doubt.

The case is particularly significant from an epistemological perspective, as it highlights the intrinsic difficulties involved in establishing causal relationships between psychotherapeutic interventions and subsequent psychopathological outcomes. Unlike somatic medicine, forensic assessment in psychology often relies on multifactorial and non-linear explanatory models, making the identification of universally accepted causal laws especially complex. The judgment therefore illustrates the tension between psychiatric-psychological expertise and criminal law standards of proof in cases involving alleged therapeutic malpractice.

## Discussion and conclusions

The assessment of testimonial reliability in criminal proceedings lies at the intersection between scientific knowledge of memory processes and the legal requirements of evidentiary fact-finding. As emerges from the theoretical framework outlined above, memory does not constitute a static repository of information, but rather a reconstructive process whose reliability is shaped by the ways in which recollection is elicited, retrieved, and reworked over time (Craik, 2020; Lacy and Stark, 2013). From this perspective, the forensic relevance of EMDR does not concern its clinical effectiveness in the treatment of trauma, which is widely recognized, but rather the ways in which the therapeutic experience may affect the processes of memory retrieval and narrative construction when such recollections are subsequently relied upon in judicial settings (Ficorilli, 2018).

Considering the most recent developments in forensic scholarship, having undergone a psychotherapeutic treatment, including EMDR, cannot be regarded as an indicator of testimonial unreliability. Rather, literature oriented toward judicial practice emphasizes the need for a cautious and non-automatic evaluative approach, focused on the concrete modalities through which the memory was formed, transformed, and documented. In this sense, attention shifts from the therapeutic technique per se to potentially relevant procedural and contextual factors, such as the presence of suggestive practices, the traceability of the clinical pathway, and the clear separation between therapeutic objectives and evidentiary fact-finding requirements, which together constitute the framework within which testimonial reliability should be assessed in the individual case (Selaya et al., 2023).

The findings of the present study add further evidence in this direction, suggesting that, within the analyzed decisions, the Italian Supreme Court showed a recurring tendency to adopt a cautious and procedurally oriented approach when addressing the alleged influence of EMDR on testimonial reliability. Judicial reasoning consistently emphasizes the need for concrete, case-specific evidence of memory distortion, rather than reliance on abstract or generalized assumptions concerning psychotherapeutic techniques. Within this framework, EMDR is recognized as a legitimate, evidence-based treatment for trauma, and concerns regarding testimonial reliability are confined to situations in which demonstrable methodological or procedural shortcomings are identified, such as suggestive practices, inadequate clinical documentation, or improper overlap between therapeutic and investigative roles.

The international literature examining the admissibility in judicial proceedings of memories that have emerged or been reworked during trauma-focused therapies, including EMDR, describes a generally non-automatic judicial approach (Otgaar et al., 2022a). Within this framework, having undergone a therapeutic treatment does not, in itself, entail the unreliability of testimony, rather it requires a more careful assessment of the modalities through which the memory was formed and transformed (Otgaar et al., 2021; Dodier et al., 2023). From this perspective, particular attention is paid to the presence of potential suggestive factors, the traceability of the clinical pathway, and the clear distinction between therapeutic objectives and evidentiary fact-finding requirements elements that recur in the literature as key criteria for scrutinizing testimonial reliability (Otgaar et al., 2022a; Selaya et al., 2023).

The relevance of these criteria becomes especially evident when considering several well-known judicial cases from the 1980s and 1990s, in which high levels of suggestibility adversely affected the quality of statements, thereby undermining their reliability. In this context, the so-called *daycare abuse cases* are frequently cited as paradigmatic examples, as they were characterized using highly suggestive interviewing practices with minors, which led to the formulation of allegations that were later shown to be unfounded (Schreiber et al., 2007).

In response to these critical issues, the literature reports that in some legal systems institutional safeguard measures have been developed to strengthen epistemic oversight in cases marked by a high risk of false memory formation. By way of example, since 1999 the Dutch Expert Committee for Equivocal Allegations of Sexual Abuse has operated as a multidisciplinary body providing technical expertise in proceedings characterized by an elevated risk of false memory formation, as well as issuing recommendations on investigative and clinical practices (Nierop et al., 2021). Taken together, these contributions outline an approach that avoids automatic evaluative assumptions and instead favors a contextual, case-by-case assessment focused on the concrete process of memory construction, rather than on the mere label of the therapeutic technique employed.

Overall, the analyzed decisions appear to reflect a cautious and context-sensitive orientation in the integration of clinical knowledge into legal reasoning, though the limited corpus of five decisions warrants caution in drawing definitive conclusions. These findings should be regarded as exploratory, providing an initial framework of the Italian jurisprudential approach rather than establishing a generalizable model. Within this framework, the Court acknowledges the reconstructive nature of memory, respects the legitimacy of therapeutic care, and simultaneously maintains stringent standards for evidentiary evaluation. Where testimonial reliability was questioned, this occurred because of identifiable methodological or procedural deficiencies, rather than any presumed intrinsic suggestiveness of EMDR as a therapeutic technique.

The present study represents, to the best of our knowledge, the first qualitative analysis of Italian case law concerning testimonial reliability following EMDR treatment. However, several limitations should be acknowledged. First, the findings may have been influenced by the restrictive eligibility criteria adopted, which may have led to the exclusion of additional potentially significant rulings. Accordingly, the observed patterns should not be interpreted as representative of Italian jurisprudence as a whole, but rather as preliminary findings emerging from the limited corpus of decisions analyzed. Second, the search was limited to a single database (DeJure) and to a targeted keyword combination, which may not have captured all relevant decisions available in the Italian legal system. Third, the small size of the analytical corpus, five decisions, limits the robustness of any pattern level conclusions. These constraints should be borne in mind when interpreting the findings, which are explicitly exploratory in nature. Notwithstanding these limitations, the study provides an initial qualitative framework of the Italian jurisprudential approach to the issue, contributing to the scientific literature and laying the groundwork for future research with broader search strategies and comparative frameworks across other jurisdictions.

In conclusion, across the analyzed cases, EMDR did not emerge as an automatic weakening factor of testimonial credibility. Rather, the decisions examined treated EMDR as a clinically legitimate intervention whose forensic relevance depended on the specific procedural and contextual circumstances of each case.

## Data Availability

The original contributions presented in the study are included in the article/supplementary material, further inquiries can be directed to the corresponding author.

## Footnotes

1 In the Italian criminal procedural system, proceedings at first instance are generally conducted before the ordinary criminal court, which may sit either as a single judge or as a panel, depending on the seriousness of the alleged offense. The first-instance trial represents the stage at which the court examines the merits of the case for the first time. Judgments rendered at first instance may be challenged by way of appeal. In appellate proceedings, judicial review is limited to the specific grounds raised in the notice of appeal: the Court of Appeal is therefore called upon to re-examine the first-instance decision and to rule anew on the case, but only within the confines of the arguments advanced by the parties. Against the judgment of the Court of Appeal, a further appeal may be lodged before the Supreme Court, (Italian Supreme Court). The Supreme Court represents the final level of adjudication and does not conduct a review on the merits. Rather, it performs a legality review, verifying the correct application and interpretation of the law. It does not reassess the facts of the case or engage in fact-finding.
